# Clinical Course, Laboratory Findings, and Tissue Distribution of Highly Pathogenic Avian Influenza A(H5N1) Virus in a Naturally Infected Domestic Cat

**DOI:** 10.3390/v18091031

**Published:** 2026-09-16

**Authors:** Agata Augustyniak, Ewelina Czyżewska-Dors, Dominik Łagowski, Katarzyna Domańska-Blicharz, Anna Lisowska, Justyna Opolska, Maciej Gogulski, Małgorzata Pomorska-Mól

**Affiliations:** 1 Department of Preclinical Sciences and Infectious Diseases, Poznan University of Life Sciences, 60-637 Poznań, Poland; agata.augustyniak@up.poznan.pl (A.A.); dlagowsky@gmail.com (D.Ł.); maciej.gogulski@up.poznan.pl (M.G.); 2 Department of Internal Diseases and Diagnostics, Poznań University of Life Sciences, 60-637 Poznań, Poland; ewelina.czyzewska-dors@up.poznan.pl; 3 Department of Virology and Viral Animal Diseases, National Veterinary Research Institute, 24-100 Puławy, Poland; domanska@piwet.pulawy.pl (K.D.-B.); anna.lisowska@piwet.pulawy.pl (A.L.); justyna.opolska@piwet.pulawy.pl (J.O.)

**Keywords:** highly pathogenic avian influenza, H5N1, infection, cat, feline, Poland

## Abstract

In June 2023, Poland experienced an unprecedented outbreak of highly pathogenic avian influenza (HPAI) H5N1 virus infections in cats, raising concerns about the role of cats in HPAI H5N1 virus ecology and potential public health implications. We describe the clinical course and molecular findings in a cat designated as cat No. 14 during this outbreak, with emphasis on the viral load detected in various organs and tissues. The cat was initially examined for recurrent dermatological problems, although the owner also reported reduced activity. Over the following five days, the disease rapidly progressed, with systemic and neurological signs unresponsive to treatment. Blood analyses revealed no major haematological abnormalities, whereas serial serum biochemistry showed progressive abnormalities. Due to the poor prognosis, the cat was humanely euthanized five days after the initial veterinary examination. Tissue samples from major internal organs and lymph nodes, together with tracheal and rectal swabs and bile, were collected for real-time RT-PCR. Viral RNA was detected in all analysed samples except the intestines, mesenteric lymph nodes, rectal swab, and bile, with the highest viral loads observed in the cerebellum and brain. This case provides molecular evidence of widespread HPAI H5N1 viral RNA distribution across tissues, complemented by detailed haematological and biochemical findings, further characterizing the systemic nature of infection.

## 1. Introduction

Highly pathogenic avian influenza (HPAI) is caused by variants of two subtypes of Influenza A Virus (IAV), H5 and H7, belonging to the *Orthomyxoviridae* family [1,2,3]. Due to its high morbidity and mortality [2], the disease severely impacts the poultry sector worldwide, causing significant economic losses estimated at billions of dollars (reviewed in [4]). The importance and danger of this disease are exemplified by the strict legal restrictions established in some countries. For example, according to the Commission Implementing Regulation (EU) 2018/1882, HPAI in birds is classified as category A, which includes diseases for which rapid eradication measures must be taken immediately upon detection [5]. All European Union (EU) member states must also maintain disease surveillance in domestic and wild birds [6]. In addition, HPAI H5N1 virus represents an increasing risk for global public health due to its zoonotic potential. This is especially true for the H5 subtype, which, until 1997, was known only as a bird pathogen. In May 1997, the first human infection with this subtype was described in Hong Kong, where a 3-year-old boy developed fatal respiratory disease [7]. According to the World Health Organisation (WHO), between 2003 and March 2026, a total of 997 human H5N1 infections worldwide were confirmed, of which 478 were fatal, indicating a high mortality rate [8]. However, transmission of HPAI from birds to mammals, including humans, has occurred sporadically to date. The “Updated joint FAO/WHO/WOAH public health assessment of recent high pathogenicity avian influenza A(H5) virus events in animals and people” states the circulating Gs/Gd-like HPAI A H5 viruses represent a low risk for global public health, simultaneously highlighting a higher risk for certain groups (e.g., occupational risk, individuals prolongedly exposed) and that this assessment can rapidly change [9]. Only within the last two centuries have there been four devastating influenza pandemics, causing hundreds of thousands, or millions of deaths [10]. All four viruses that caused these pandemics were of animal origin, mainly avian. The H2N2/1957 and H3N2/1968 are believed to be reassortants of human and avian viruses; meanwhile, A/H1N1pdm09 is a triple reassortant of human, avian, and swine IAVs [10]. The precise origin of the H1N1/1918 virus remains unclear; however, one of the most recent approaches supports the hypothesis of reassortment between human and avian IAVs [11]. Regarding influenza infections, pigs are the best-known example of “mixing vessels”, a host in which potential reassortments can occur, as they can be simultaneously infected with multiple IAVs, like human and avian [2]. Available data indicate that other animal species can also serve as such “mixing vessels”, including companion animals (reviewed in [2,12]). Recent reports indicate the presence of sialic acid receptors that bind both avian and human influenza viruses, potentially enabling the emergence of new reassortants, including in cats [13,14]. The emergence of reassortants increases the risk of virus establishment, which can be easily transmitted between mammals, including humans.

By 2021, HPAI H5N1 virus had become the predominant subtype within clade 2.3.4.4b, contributing to an ongoing HPAI panzootic [15,16]. To date, infections with this clade or its reassortants have been reported worldwide (reviewed in [15]). In addition, the clade 2.3.4.4b H5N1 seems to have an unusual feature: more frequent spillover to various mammal species and subsequent cases of mammal-to-mammal transmission, which is alarming [15,16]. In light of these findings, many authors have emphasised the need to strengthen HPAI surveillance not only in birds but also in mammals [14,15,16,17,18,19].

Domestic cats are among the most commonly kept companion animals worldwide. In Poland, approximately 40–41% of households own at least one cat, placing Poland among the leading European countries in cat ownership [20]. Given their close and frequent contact with humans, feline H5N1 infections are of particular importance from both veterinary and public health perspectives. Cats may act as an epidemiological interface between wildlife and humans: many live in close contact with their owners while simultaneously having unsupervised outdoor access, increasing the risk of exposure to infected wild animals and the transmission of zoonotic pathogens. Sporadic cases of HPAI infection in cats have been reported since 2004 [1], and domestic cats are among the mammalian species most frequently affected by the H5N1 virus of clade 2.3.4.4b. In 2023, an unprecipitated HPAI outbreak including more than 20 cats was noted in Poland [21,22,23]. Detailed characteristics of the virus revealed that it belonged to the CH genotype (H5N1 A/Eurasian wigeon/Netherlands/3/2022-like), which was circulating at that time in poultry and wild birds in Europe. Although poultry raw meat appeared to be one of possible sources of infection, cats that were not fed such meat but had unrestricted access to the outdoors were also infected. The worrying number of cats with HPAI H5N1 virus infection in Poland caused the inclusion of this species in the “National Programme for the surveillance of animals for avian influenza for 2025–2027” [24]. In addition, some mutations indicative of viral adaptation to mammals were identified in cats infected with HPAI H5N1 virus [14,18,21,22,25]. Available data show that infection in cats has a mostly severe, rapid course that often leads to death. The recent meta-analysis showed an emerging role for cats in the ecology of HPAI [17], and some authors suggest that cats may contribute to HPAI H5N1 virus spillovers [14,26]. Although definitive evidence of HPAI H5N1 virus transmission from cats to humans remains lacking, recent reports suggest that such transmission may be possible. Serological evidence of HPAI H5N1 virus infection was detected in a veterinarian exposed to an infected cat [27]. Taking all the aforementioned into account, there is an urgent need for a better understanding of this infection. Therefore, the notified cases should be carefully investigated. This paper presents the clinical course of the disease in cat No. 14 infected with the H5N1 HPAI virus during the 2023 outbreak in Poland.

## 2. Detailed Case Description

### 2.1. Case Presentation

We have described herein in detail the case of a cat, which was confirmed to be HPAI H5N1 virus-positive as presented in the previous report (cat no. 14 in [21]). The cat was an eight-year-old male weighing 6.9 kg, fed a raw poultry-based diet, and with outdoor access. Importantly, another cat in the same household remained clinically healthy; however, it was fed only a commercial diet (without raw meat) and kept strictly indoors.

On 15 June 2023, the cat was presented to the veterinary clinic for a follow-up examination of recurrent dermatological lesions, as recommended by the attending veterinarian during a previous visit approximately one year earlier. According to the owner, the cat had exhibited lethargy during the preceding week, although its appetite remained normal.

Physical examination revealed recurrent skin lesions involving the lips and abdomen, which were markedly exacerbated at the time of presentation, as well as a pronounced enlargement of the right submandibular lymph node. The cat was alert and responsive, with an appropriate response to external stimuli. The pulse was well-defined. The abdomen was non-tense and non-painful on palpation. Thoracic auscultation revealed normal respiratory sounds and regular, strong cardiac sounds without detectable abnormalities. The rectal temperature was 38.2 °C. A complete blood count revealed changes in several parameters, including a marked increase in eosinophil and basophil concentration and percentage, a mild leukocytosis, and a decreased neutrophil percentage (Table 1). Serum biochemistry showed an increased lipase concentration, while total thyroxine, urea, triglycerides, and the albumin-to-globulin ratio were at the upper limits of the reference intervals (Table 2). The tentative clinical diagnosis was dermatitis and/or pharyngitis. No treatment was initiated at that visit, although further medical management, including antimicrobial therapy, was considered if clinically indicated.

Two days later, on 17 June 2023, the cat was re-presented to the veterinary clinic due to deterioration in its clinical condition. According to the owner, the cat was markedly less active, spent most of the time sleeping, and was unwilling to go outdoors. Physical examination revealed pyrexia (40.2 °C), pronounced enlargement of the right submandibular lymph node compared with the previous examination, and pharyngeal erythema. The mucous membranes were pink and moist, and the capillary refill time was <2 s. The abdomen was soft and non-painful on palpation. Thoracic auscultation revealed normal respiratory sounds and a regular cardiac rhythm with strong heart sounds, and the pulse was of normal quality. A tentative diagnosis of pharyngitis was maintained. No additional diagnostic tests were performed at this visit. Treatment consisted of injectable tolfenamic acid and cefovecin.

On 18 June 2023, the cat was re-examined because no clinical improvement had been observed. According to the owner, the cat remained lethargic, slept most of the time, refused to drink, and had experienced several episodes of vomiting. Physical examination revealed a rectal temperature of 38.6 °C. The cat remained responsive to external stimuli. Previously enlarged lymph nodes were no longer palpably enlarged. The mucous membranes were not reported as abnormal. The abdomen was soft and non-painful on palpation, the pulse was of normal quality, and thoracic auscultation revealed normal respiratory sounds and a regular cardiac rhythm with strong heart sounds.

Haematological examination showed no clinically relevant abnormalities (Table 1). However, serum amyloid A concentration was markedly elevated at 181 mg/L (Table 2). A tentative diagnosis of inflammation and/or pharyngitis was maintained. Treatment included intravenous administration of a balanced multi-electrolyte solution, maropitant, and buprenorphine.

Two days later, on 20 June 2023, the cat’s condition had deteriorated further. According to the owner, no clinical improvement had been observed, and the cat had become progressively weaker and anorectic. Physical examination revealed marked hypothermia (35.0 °C), reduced responsiveness to environmental stimuli, and icteric mucous membranes. Serum amyloid A concentration remained markedly elevated (191 mg/L). Owing to the severity of the clinical condition, the cat was hospitalised. During hospitalisation, epileptic seizures developed, and the cat progressed to an agonal state. As the cat did not respond to the implemented treatment and the prognosis was poor, humane euthanasia was performed. Haematological examination revealed no major abnormalities (Table 1). In contrast, serum biochemistry showed severe abnormalities, including marked increases in alanine aminotransferase, bilirubin, urea, blood urea nitrogen, creatinine, and phosphorus, and decreases in total protein and calcium (Table 2). These findings were consistent with severe multisystemic disease involving hepatic and renal dysfunction. At this stage, a viral disease was suspected. Following euthanasia, and with the owner’s consent, the carcass was frozen pending sample collection for further diagnostic investigations. Therefore, a complete necropsy and histopathological examination were not performed.

At that time, information regarding an increasing number of fatal disease cases in cats, characterised by neurological and respiratory signs, was being reported by veterinary practitioners and cat owners in Poland. Consequently, throat swabs were taken post-mortem and sent to the National Reference Laboratory for Avian Influenza in the National Veterinary Research Institute in Puławy (Poland) for testing for HPAIV, where a high viral load with Ct values of 19.8, 20.6 and 20.7 for M, H5 and N1 genes, respectively, was detected [21]. Therefore, samples of internal organs (brain, cerebellum, heart, lung, kidney, liver, spleen, stomach, duodenum, small and large intestine, mesenteric, submandibular, subscapular, tracheobronchial, and retropharyngeal lymph nodes), bile, and swabs (tracheal and rectal) were additionally collected and stored in deep freeze for further investigation.

### 2.2. Determination of Virus Quantity

Samples of tissues/organs collected from the dead cat during necropsy were homogenised in PBS to 20% (*w*/*v*) suspension and centrifuged (3500 *g*, 10 min). RNA extraction from the obtained supernatants was performed using the IndiMag automated extraction technology with the dedicated IndiMag Pathogen Kit (Indical BioScience, Leipzig, Germany) according to the manufacturer’s instructions. The quantity of viral RNA was measured by one-step real-time RT-PCR using primers and a probe specific to the influenza M gene [29]. Reactions were performed using QuantiTect Probe RT-PCR Kit (Qiagen, Hilden, Germany) in 7500 Real Time PCR System (Applied Biosystems, Waltham, MA, USA) with the following protocol: 50 °C for 30 min and 95 °C for 15 min, followed by 40 cycles of 95 °C for 10 s and 60 °C for 30 s. Ten-fold dilutions of the virus suspension with a known EID50 titer were tested in triplicate, in parallel with the cat samples to generate the standard curve. The amplification efficiency, coefficient of determination, and limit of detection for the real-time RT-PCR test were also calculated. The quantity of viral RNA was expressed as equivalents of EID50 (eqEID50) in 1 g of tissue.

**Figure 1:**
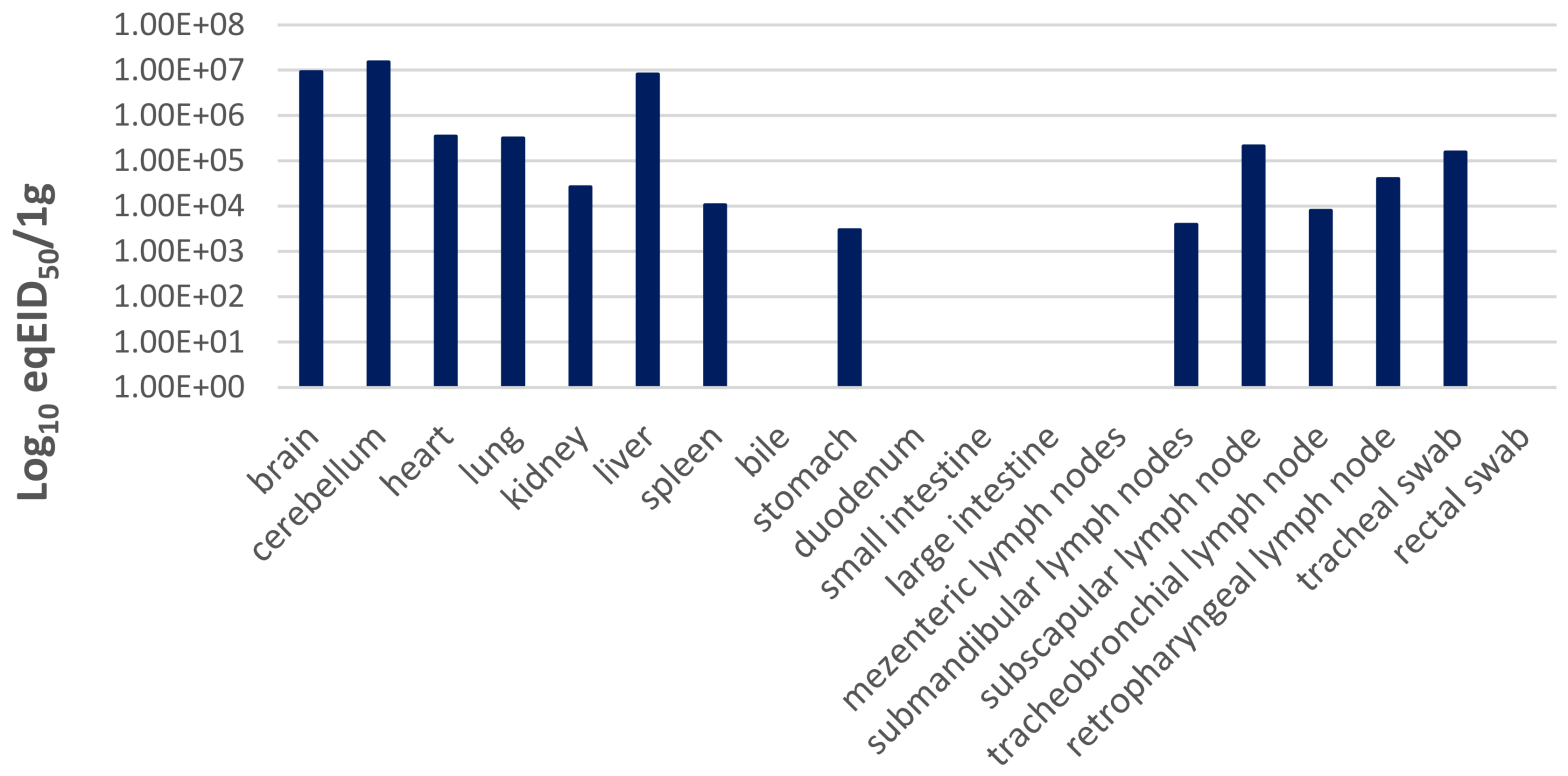
Viral loads of HPAI H5N1 virus in different samples.

The standard curve performed exhibited acceptable parameters, with a slope of −3.562, a coefficient of determination (R^2^) of 0.999, and an amplification efficiency of 90.882. The limit of detection was set at 1.6 × 10^3^ EID_50_. Of the nineteen samples (tissues and organs) collected post mortem, viral RNA was detected in 13, with viral loads ranging from 10^3^ to 10^7^ EID_50_ equivalents (Figure 1). No viral RNA was detected in the duodenum, small intestine, large intestine, bile, rectal swab, or in the mesenteric lymph nodes draining the gastrointestinal tract. Interestingly, viral RNA was detected in the stomach at a level corresponding to 3.02 × 10^3^ EID_50_ equivalents, despite the absence of detectable viral RNA in the intestines and associated mesenteric lymph nodes. The highest viral loads were detected in the central nervous system, particularly in the brain and cerebellum, and in the liver, where levels reached 10^6^–10^7^ EID_50_ equivalents.

## 3. Discussion

The etiological agent of infection described herein was determined to be influenza A virus, H5N1 clade 2.3.4.4b, genotype CH (H5N1_A/Eurasian_Wigeon/Netherlands/3/2022-like), and its detailed characteristics were previously reported by Domańska-Blicharz et al. (2023) [21]. Because the infected cat was fed raw poultry meat and had outdoor access, two potential sources of infection were primarily considered: contaminated poultry meat and exposure to infected wild birds and/or their faeces. Notably, the second cat, who never went outdoor nor ate raw meat, living in the same household remained clinically healthy throughout the observation period, suggesting that either there was no direct transmission of the virus between the cats, or the infectious dose was too low to cause disease. During the June 2023 disease outbreak, cases in cats were recorded concurrently across multiple distinct regions of Poland; subsequently, new reports ceased. The viruses detected in cats during the outbreak showed a high degree of similarity among themselves and were similar to viruses circulating in wild birds and poultry at that time in Poland, which may indicate that the cats were exposed to infected birds or a contaminated environment [21]. On the other hand, viral genetic material was detected in one of the five chicken meat samples tested, which came from the same households as an ill cats, and although this sample came from the private fridge and it cannot be ruled out that it may have become contaminated after slaughter, during transport or in the household, this was also a possible source of infection for the cats [22]. For a more detailed discussion of the potential sources of feline infection during the outbreak, see [21].

In this case, it is difficult to precisely estimate the interval between the onset of clinical signs and death. The observed decrease in activity during the week preceding the first veterinary examination was not the primary reason for the visit. At the initial examination, the predominant findings included recurrent dermatological lesions and enlargement of the right submandibular lymph node, both of which could reasonably be interpreted in the context of the cat’s previous history of recurrent skin disease. However, the time from the first veterinary examination to euthanasia was only 5 days, indicating a very rapid disease course. This interval was slightly longer compared with the survival time reported in multiple previously described cases [17,30]. The clinical symptoms observed in the case described herein are in line with those observed in other cats with confirmed H5N1 infection during the 2023 outbreak, with rapid progression from nonspecific clinical signs to severe disease and neurological involvement [21,30,31]. However, neurological manifestations developed relatively late in the present case compared with those reported previously. Jańczak et al. (2025) reported that neurological signs appeared within 24 h of disease onset in many affected cats [30]. In addition, respiratory involvement was less pronounced than in most cases reported during the outbreak. Nonspecific signs, including lethargy, reduced activity, anorexia, and pyrexia, dominated the initial clinical picture. In contrast, neurological manifestations became apparent only at an advanced stage of disease, shortly before euthanasia.

In the present case, viral RNA was detected in all examined organs except the intestines and regional mesenteric lymph nodes (Figure 1), suggesting widespread dissemination of the virus within the affected animal. However, the detection of viral RNA by RT-qPCR alone is insufficient to draw definitive conclusions regarding viral replication or tissue tropism. Such conclusions should be supported by histopathological and immunohistochemical findings. Nevertheless, the results of the present study appear to support previous observations reported in the literature. The most common clinical manifestations of HPAI infection in domestic cats include respiratory and neurological signs, as well as systemic disease [14,18,21,22,30,32,33,34]. Therefore, the detection of high viral loads in tissues of the respiratory and nervous systems was not unexpected. The highest viral loads were detected in the cerebellum and brain, consistent with findings from previous studies [14,19,32,35], whereas viral loads detected in the lung tissue and tracheal swab were lower. These findings support Chothe et al.’s (2025) observation of enhanced neurotropism of the H5N1 clade 2.3.4.4b [14]; however, the mechanism driving this shift remains unknown. We also found relatively high viral loads in the liver compared to the respiratory samples. Similarly, low Ct values in the liver have been reported in other studies [25,31,32,36]. In one case report, liver disease was suspected to be an important factor in HPAI pathogenesis in the evaluated dead cats, as a strong viral antigen signal on immunohistochemistry overlapped with histopathological changes [37]. The recent meta-analysis indicates the liver as one of the organs in which pathological changes during necropsy are most commonly detected [17]. Vahlenkamp et al. (2010) suggest that severe liver damage during HPAI H5N1 virus infection indicates that the liver may represent an important target organ in cats [36]. The high viral RNA loads detected in the liver in the present case support this hypothesis. Interesting results were obtained for digestive tract samples: viral genetic material was absent in all samples except the stomach, where the lowest viral loads were observed. In previous reports, HPAI H5N1 viral genetic material was detected in the intestinal samples collected from infected cats [14,25,31]. The absence of detectable viral RNA in the intestines may be related to the limited stability of influenza A viruses under acidic gastrointestinal conditions, although this hypothesis requires further investigation [38]. Under experimental conditions, changes in pH were shown to affect IAV viability [39,40,41]. Nevertheless, H5N1 virus replication in intestinal tissues has been reported in both humans and animals, indicating that gastrointestinal infection can occur under certain circumstances [42]. Although oral exposure is considered the most likely route of infection in the present case, the precise route of virus entry cannot be indicated. It cannot be excluded that viral entry occurred through the oral, pharyngeal, or upper respiratory mucosa before widespread dissemination of the infection. In experimental infection studies, in some cats the viral genetic material was detected in gastrointestinal samples following intratracheal, oculo-nasopharyngeal, oral, and intravenous inoculations, whereas in others it was absent [36,43].

Contrary to some previous reports [30], and in line with another one [31], no major haematological abnormalities were observed throughout the course of the disease. The only notable haematological abnormalities were a mild relative neutrophilia and eosinopenia, findings consistent with a stress leukogram but also commonly observed in systemic inflammatory conditions. The marked eosinophilia and basophilia observed in the initial haematological examination may have been associated with the cat’s history of recurrent dermatological disease and the exacerbation of skin lesions noted at presentation. Alternative causes of eosinophilia, including parasitic infestation, cannot be excluded; however, no diagnostic data regarding parasitological status were available for review. In the present case, the markedly elevated serum amyloid A concentration has been observed. Similar findings have been reported for other IAV infections in other multiple species [44,45,46]. The biochemical abnormalities indicated severe dysfunction of multiple organ systems. In particular, markedly elevated alanine aminotransferase activity and bilirubin concentration were consistent with previous reports [30,31,36] and suggested severe liver injury. Marked elevations in creatinine, urea, blood urea nitrogen, and phosphorus concentrations suggested substantial renal involvement. Interestingly, this finding contrasts with the results reported by Jańczak et al. (2025), in which renal biochemical parameters remained within reference intervals in most evaluated cats during the 2023 outbreak [30]. A progressive decrease in total protein, albumin, and globulin concentrations was observed during the 5-day course of illness. This decline may have been associated with the severe systemic inflammatory response, as albumin is a negative acute-phase protein and its synthesis decreases during inflammation [47]. Concurrent severe hepatic and renal injury may also have contributed to the altered protein concentrations. However, the relative contributions of these mechanisms cannot be determined from the available data.

The results presented above provide additional detailed information on H5N1 HPAI virus diagnostics in cats. For testing in live animals, respiratory samples, such as nasopharyngeal or pharyngeal swabs, should be considered first. However, obtaining such a sample typically requires sedation or general anaesthesia, which, in patients in severe condition, carries a substantially elevated risk of complications. Some studies detected viral genetic material in rectal swabs or faeces [25,43], contradicting our results; in others, rectal swab samples tested negative by PCR [18,32]. Our findings, although based on a single animal, confirm previous observations that a negative rectal swab result should be interpreted with caution, as viral RNA may be undetectable in this specimen despite systemic infection.

## 4. Conclusions

Given the growing number of H5N1 infections in cats worldwide, their severe clinical course, and reports of mutations associated with mammalian adaptation, further research is urgently needed to better understand the epidemiology, pathogenesis, transmission dynamics, and diagnostic aspects of this emerging disease. Furthermore, recent reports raising the possibility of cat-to-human transmission highlight the importance of a One Health approach [27]. Therefore, awareness should be raised among cat owners, veterinarians, and others in close contact with cats. In regions where HPAI viruses are circulating among domestic poultry and wild birds, HPAI H5N1 virus infection should be included in the differential diagnosis of cats presenting with acute neurological and/or respiratory signs, particularly when accompanied by pyrexia, lethargy, or anorexia. The results presented provide valuable insight into the distribution of the virus in infected cats.

## Figures and Tables

**Table 1:** Haematological results.

Parameter	June 15th	RI ^a^	June 18th	June 20th	RI ^a^
White blood cells (G/L)	↑ **11.5**	6–11	6	7.09	5.5–19.5
Neutrophils (G/L)	5.75	3–11	4.94	5.87	3.12–12.58
Neutrophils %	↓ **49.8**	60–78	↑ **82.2**	↑ **82.7**	38–80
Lymphocytes (G/L)	3.3	1–4	0.85	0.95	0.73–7.86
Lymphocytes %	28.6	15–38	14.1	13.3	12–45
Monocytes (G/L)	0.12	0.04–0.5	0.07	0.14	0.07–1.36
Monocytes %	1	0.01–4	1.3	2	1–8
Eosinophils (G/L)	↑ **1.69**	0.04–0.6	0.11	↓ **0.04**	0.06–1.93
Eosinophils %	↑ **14.7**	0.01–6	1.9	↓ **0.6**	1–11
Basophils (G/L)	↑ **0.679**	0.001–0.1	0.03	0.09	0–0.12
Basophils %	↑ **5.9**	0.001–1	0.5	↑ **1.4**	0–1.2
Red blood cells (T/L)	9.7	5–10	↑ **10.27**	10.12	4.62–10.2
Haemoglobin (g/L)	136	90–150	144	143	85–153
Haematocrit (L/L)	0.431	0.3–0.044	0.433	0.413	0.26–0.47
Mean Corpuscular Volume (fL)	44.4	40–55	42.2	40.9	38–54
Mean Corpuscular Haemoglobin (pg)	14	13–16	14	14.2	11.8–18
Mean Corpuscular Haemoglobin Concentration (g/L)	316	310–360	333	347	290–360
Platelets (G/L)	192	180–550	202	179	100–518

RI—reference intervals; ↑ above the reference interval; ↓ below the reference interval; bold—values outside RI; ^a^ Reference intervals correspond to the diagnostic laboratory that performed the analysis.

**Table 2:** Serum biochemistry results.

Parameter	June 15th	RI ^a^	June 18th	June 20th	RI ^a^
Albumin (g/L)	38.5	26–46	NT	28	25–46
Globulin (g/L)	34.6	19–66	NT	27	26–51
Albumin/globulin ratio (A/G ratio)	↑ **1.11**	0.5–1.1	NT	1	
Alanine aminotransferase (U/L)	46.7	1–91	NT	↑ **1100**	0–116
Alpha-amylase (U/L)	790	10–1850	NT	1264	500–1600
Aspartate aminotransferase (U/L)	23.9	1–59	NT	NT	
Total protein (g/L)	73.1	57–94	NT	↓ **55**	57–89
Total bilirubin (µmol/L)	1.78	0.01–5.5	NT	↑ **114.6**	0–15.4
Cholesterol (mmol/L)	3.31	1.8–6.5	NT	3.93	1.4–5.7
Creatine kinase (U/L)	133	50–690	NT	NT	
Glutamate dehydrogenase (U/L)	4.34	0.01–6	NT	NT	
Glucose (mmol/L)	5.38	3.05–6.1	NT	6.27	2.94–8.33
Gamma-glutamyl transferase (U/L)	1.01	0.01–5	NT	10	0–10
Creatinine (µmol/L)	110.3	1–168	NT	↑ **394.26**	61.9–176.8
Lactate dehydrogenase (U/L)	128	0.1–606	NT	NT	
Urea (mmol/L)	↑ **11.7**	5–11.3	NT	↑ **27**	4.6–13.2
Blood urea nitrogen (mg/dL)	NT		NT	↑ **75.9**	13–37
Triglycerides (mmol/L)	↑ **1.22**	0.06–1.14	NT	NT	
Chloride (mmol/L)	119.4	110–130	NT	NT	
Magnesium (mmol/L)	0.88	0.6–1.3	NT	NT	
Potassium (mmol/L)	4.35	3–4.8	NT	NT	
Sodium (mmol/L)	156	145–158	NT	NT	
Calcium (mmol/L)	2.57	2.3–3	NT	↓ **1.3**	2–3
Alkaline phosphatase (U/L)	30	1–140	NT	52	0–111
Phosphorus (mmol/L)	1.32	0.8–1.9	NT	↑ **3.5**	1–2.4
Lipase (U/L)	↑ **34.2**	0–26	NT	25	0–35
Fructosamine (µmol/L)	212.3	160–335	NT	NT	
Total thyroxine (T4) (µg/dL)	↑ **2.73**	1–2.5	NT	NT	
Serum amyloid A (mg/L)	NT		↑ **181**	↑ **191**	~0 (based on [28])

RI—reference intervals; ↑ above the reference interval; ↓ below the reference interval; bold—values outside RI; ^a^ Reference intervals correspond to the diagnostic laboratory that performed the analysis.

## Data Availability

The original contributions presented in this study are included in the article. Further inquiries can be directed to the corresponding author.

## Abbreviations

The following abbreviations are used in this manuscript:

IAV	Influenza A virus
HPAI	Highly pathogenic avian influenza
AI	Avian influenza
RT-PCR	Reverse transcription-polymerase chain reaction

## References

[1] Marschall J., Hartmann K. (2008). Avian influenza A H5N1 infections in cats. J. Feline Med. Surg..

[2] Abdelwhab E.M., Mettenleiter T.C. (2023). Zoonotic Animal Influenza Virus and Potential Mixing Vessel Hosts. Viruses.

[3] Graziosi G., Lupini C., Catelli E., Carnaccini S. (2024). Highly Pathogenic Avian Influenza (HPAI) H5 Clade 2.3.4.4b Virus Infection in Birds and Mammals. Animals.

[4] Nisaa Z., Häsler B., Bennani H., Jorquera R., Scolamacchia F., Alarcon P. (2026). Global economic impacts of Highly Pathogenic Avian Influenza: A systematic review and impact framework. Prev. Vet. Med..

[5] Commission Implementing Regulation (EU) 2018/1882 of 3 December 2018, on the Application of Certain Disease Prevention and Control Rules to Categories of Listed Diseases and Establishing a List of Species and Groups of Species Posing a Considerable Risk for the Spread of Those Listed Diseases. https://eur-lex.europa.eu/eli/reg_impl/2018/1882/oj/eng.

[6] Commission Implementing Regulation (EU) 2020/690 of 17 December 2019, Laying Down Rules for the Application of Regulation (EU) 2016/429 of the European Parliament and of the Council as Regards the Listed Diseases Subject to Union Surveillance Programmes, the Geographical Scope of Such Programmes and the Listed Diseases for Which the Disease-Free Status of Compartments May Be Established. https://eur-lex.europa.eu/eli/reg_impl/2020/690/oj/eng.

[7] Subbarao K., Klimov A., Katz J., Regnery H., Lim W., Hall H., Perdue M., Swayne D., Bender C., Huang J. (1998). Characterization of an avian influenza A (H5N1) virus isolated from a child with a fatal respiratory illness. Science.

[8] Cumulative Number of Confirmed Human Cases for Avian Influenza A(H5N1) Reported to WHO, 2003–2026, 31 March 2026. https://www.who.int/publications/m/item/cumulative-number-of-confirmed-human-cases-for-avian-influenza-a(h5n1)-reported-to-who--2003-2026--31-march-2026.

[9] Updated joint FAO/WHO/WOAH Public Health Assessment of Recent High Pathogenicity Avian Influenza A(H5) Virus Events in Animals and People. https://www.who.int/publications/m/item/updated-joint-fao-who-woah-public-health-assessment-of-recent-influenza-a(h5)-virus-events-in-animals-and-people-july2025.

[10] Guan Y., Vijaykrishna D., Bahl J., Zhu H., Wang J., Smith G.J. (2010). The emergence of pandemic influenza viruses. Protein Cell.

[11] Worobey M., Han G.Z., Rambaut A. (2014). Genesis and pathogenesis of the 1918 pandemic H1N1 influenza A virus. Proc. Natl. Acad. Sci. USA.

[12] Lee C.Y. (2024). Exploring Potential Intermediates in the Cross-Species Transmission of Influenza A Virus to Humans. Viruses.

[13] Wang H., Wu X., Cheng Y., An Y., Ning Z. (2013). Tissue distribution of human and avian type sialic acid influenza virus receptors in domestic cat. Acta Vet. Hung..

[14] Chothe S.K., Srinivas S., Misra S., Nallipogu N.C., Gilbride E., LaBella L., Mukherjee S., Gauthier C.H., Pecoraro H.L., Webb B.T. (2025). Marked neurotropism and potential adaptation of H5N1 clade 2.3.4.4.b virus in naturally infected domestic cats. Emerg. Microbes Infect..

[15] Chrzastek K., Lieber C.M., Plemper R.K. (2025). H5N1 Clade 2.3.4.4b: Evolution, Global Spread, and Host Range Expansion. Pathogens.

[16] Plaza P.I., Gamarra-Toledo V., Euguí J.R., Lambertucci S.A. (2024). Recent Changes in Patterns of Mammal Infection with Highly Pathogenic Avian Influenza A(H5N1) Virus Worldwide. Emerg. Infect. Dis..

[17] Bonilla-Aldana D.K., Bonilla-Aldana J.L., Acosta-España J.D., Rodriguez-Morales A.J. (2025). Highly Pathogenic Avian Influenza H5N1 in Cats (Felis catus): A Systematic Review and Meta-Analysis. Animals.

[18] Briand F.X., Souchaud F., Pierre I., Beven V., Hirchaud E., Hérault F., Planel R., Rigaudeau A., Bernard-Stoecklin S., Van der Werf S. (2023). Highly Pathogenic Avian Influenza A(H5N1) Clade 2.3.4.4b Virus in Domestic Cat, France, 2022. Emerg. Infect. Dis..

[19] Burrough E.R., Magstadt D.R., Petersen B., Timmermans S.J., Gauger P.C., Zhang J., Siepker C., Mainenti M., Li G., Thompson A.C. (2024). Highly Pathogenic Avian Influenza A(H5N1) Clade 2.3.4.4b Virus Infection in Domestic Dairy Cattle and Cats, United States, 2024. Emerg. Infect. Dis..

[20] Cat Ownership in the European Union 2023, by Country. https://www.statista.com/statistics/515464/cat-ownership-european-union-eu-by-country/?srsltid=AfmBOopPgni9Dtd4EMuC6MT9JKVIdcSlsJbxvdCzrucq1Ih6QT21h57D.

[21] Domańska-Blicharz K., Świętoń E., Świątalska A., Monne I., Fusaro A., Tarasiuk K., Wyrostek K., Styś-Fijoł N., Giza A., Pietruk M. (2023). Outbreak of highly pathogenic avian influenza A(H5N1) clade 2.3.4.4b virus in cats, Poland, June to July 2023. Euro Surveill..

[22] Rabalski L., Milewska A., Pohlmann A., Gackowska K., Lepionka T., Szczepaniak K., Swiatalska A., Sieminska I., Arent Z., Beer M. (2023). Emergence and potential transmission route of avian influenza A (H5N1) virus in domestic cats in Poland, June 2023. Euro Surveill..

[23] Disease Outbreak News Influenza A(H5N1) in Cats—Poland. https://www.who.int/emergencies/disease-outbreak-news/item/2023-DON476.

[24] Regulation of the Minister of Agriculture and Rural Development No. 1 of 19 March 2025 on the Implementation of the “National Programme for the Surveillance of Animals for Avian Influenza” for the Years 2025–2027. https://dziennikustaw.gov.pl/D2025000043501.pdf.

[25] Kang Y.M., Heo G.B., An S.H., Lee H., Park E., Cha R.M., Jang Y.Y., Sagong M., Kim A.Y., Kim J. (2024). Highly Pathogenic Avian Influenza A(H5N1) Virus Infection in Cats, South Korea, 2023. Emerg. Infect. Dis..

[26] Chen C., Naru A., Mareddy V.R., Lanka S., Olmstead C., Revindran-Stam V., Sherman M., Yee H., Loose N., Delaney M.A. (2025). Highly pathogenic avian influenza A H5N1 virus infection in an immunocompromised domestic cat. ASM Case Rep..

[27] Vaughan A., Joyce A., Traub E., Jae M., Beeler E., Paiva E., Ananian K., Holiday C., Jefferson S., Richardson J. (2026). Serologic Evidence of Highly Pathogenic Avian Influenza A(H5N1) Virus Infection in a Veterinary Professional Exposed to an Infected Domestic Cat—Los Angeles County, California, December 2024–January 2025. MMWR Morb. Mortal. Wkly. Rep..

[28] Yuki M., Aoyama R., Nakagawa M., Hirano T., Naitoh E., Kainuma D. (2020). A Clinical Investigation on Serum Amyloid A Concentration in Client-Owned Healthy and Diseased Cats in a Primary Care Animal Hospital. Vet. Sci..

[29] Nagy A., Vostinakova V., Pirchanova Z., Cernikova L., Dirbakova Z., Mojzis M., Jirincova H., Havlickova M., Dan A., Ursu K. (2010). Development and evaluation of a one-step real-time RT-PCR assay for universal detection of influenza A viruses from avian and mammal species. Arch. Virol..

[30] Jańczak D., Golke A., Szymański K., Hallmann E., Pancer K., Masny A., Dzieciątkowski T., Szaluś-Jordanow O. (2026). Clinical and Laboratory Findings in Cats with Confirmed Avian Influenza A/H5N1 Virus Infection During the 2023 Outbreak in Poland: A Retrospective Case Series of 22 Cats. Pathogens.

[31] Szaluś-Jordanow O., Golke A., Dzieciątkowski T., Chrobak-Chmiel D., Rzewuska M., Czopowicz M., Sapierzyński R., Kardas M., Biernacka K., Mickiewicz M. (2023). A Fatal A/H5N1 Avian Influenza Virus Infection in a Cat in Poland. Microorganisms.

[32] Sillman S.J., Drozd M., Loy D., Harris S.P. (2023). Naturally occurring highly pathogenic avian influenza virus H5N1 clade 2.3.4.4b infection in three domestic cats in North America during 2023. J. Comp. Pathol..

[33] Songserm T., Amonsin A., Jam-on R., Sae-Heng N., Meemak N., Pariyothorn N., Payungporn S., Theamboonlers A., Poovorawan Y. (2006). Avian influenza H5N1 in naturally infected domestic cat. Emerg. Infect. Dis..

[34] Gomez J.F., Bemis I.G., Shittu I., Gray G.C., Coleman K.K. (2025). Outbreak of highly pathogenic avian influenza A(H5N1) among house cats: A case series involving oseltamivir treatment. One Health.

[35] Mainenti M., Siepker C., Magstadt D.R., Gauger P., Baum D., Petersen B., Aubrey T., Sett K., Burrough E.R. (2025). Distribution of lesions and detection of influenza A(H5N1) virus, clade 2.3.4.4b, in ante- and post-mortem samples from naturally infected domestic cats on U.S. dairy farms. J. Vet. Diagn. Investig..

[36] Vahlenkamp T.W., Teifke J.P., Harder T.C., Beer M., Mettenleiter T.C. (2010). Systemic influenza virus H5N1 infection in cats after gastrointestinal exposure. Influenza Other Respir. Viruses.

[37] Klopfleisch R., Wolf P.U., Uhl W., Gerst S., Harder T., Starick E., Vahlenkamp T.W., Mettenleiter T.C., Teifke J.P. (2007). Distribution of lesions and antigen of highly pathogenic avian influenza virus A/Swan/Germany/R65/06 (H5N1) in domestic cats after presumptive infection by wild birds. Vet. Pathol..

[38] Hirose R., Nakaya T., Naito Y., Daidoji T., Watanabe Y., Yasuda H., Konishi H., Itoh Y. (2017). Mechanism of Human Influenza Virus RNA Persistence and Virion Survival in Feces: Mucus Protects Virions From Acid and Digestive Juices. J. Infect. Dis..

[39] Zou S., Guo J., Gao R., Dong L., Zhou J., Zhang Y., Dong J., Bo H., Qin K., Shu Y. (2013). Inactivation of the novel avian influenza A (H7N9) virus under physical conditions or chemical agents treatment. Virol. J..

[40] Wanaratana S., Tantilertcharoen R., Sasipreeyajan J., Pakpinyo S. (2010). The inactivation of avian influenza virus subtype H5N1 isolated from chickens in Thailand by chemical and physical treatments. Vet. Microbiol..

[41] Lu H., Castro A.E., Pennick K., Liu J., Yang Q., Dunn P., Weinstock D., Henzler D. (2003). Survival of avian influenza virus H7N2 in SPF chickens and their environments. Avian Dis..

[42] Shu Y., Li C.K., Li Z., Gao R., Liang Q., Zhang Y., Dong L., Zhou J., Dong J., Wang D. (2010). Avian influenza A(H5N1) viruses can directly infect and replicate in human gut tissues. J. Infect. Dis..

[43] Rimmelzwaan G.F., van Riel D., Baars M., Bestebroer T.M., van Amerongen G., Fouchier R.A., Osterhaus A.D., Kuiken T. (2006). Influenza A virus (H5N1) infection in cats causes systemic disease with potential novel routes of virus spread within and between hosts. Am. J. Pathol..

[44] Pomorska-Mól M., Markowska-Daniel I., Kwit K., Czyżewska E., Dors A., Rachubik J., Pejsak Z. (2014). Immune and inflammatory response in pigs during acute influenza caused by H1N1 swine influenza virus. Arch. Virol..

[45] Hultén C., Sandgren B., Skiöldebrand E., Klingeborn B., Marhaug G., Forsberg M. (1999). The acute phase protein serum amyloid A (SAA) as an inflammatory marker in equine influenza virus infection. Acta Vet. Scand..

[46] Vollmer A.H., Gebre M.S., Barnard D.L. (2016). Serum amyloid A (SAA) is an early biomarker of influenza virus disease in BALB/c, C57BL/2, Swiss-Webster, and DBA.2 mice. Antivir. Res..

[47] Ceron J.J., Eckersall P.D., Martýnez-Subiela S. (2005). Acute phase proteins in dogs and cats: Current knowledge and future perspectives. Vet. Clin. Pathol..

